# Microvascular density and tumor budding in oral squamous cell carcinoma

**DOI:** 10.4317/medoral.25640

**Published:** 2022-12-24

**Authors:** Eliene Magda de Assis, Mayara Rodrigues, Jéssica Campos Vieira, Maria Inês Mantuani Pascoaloti, Helvécio Marangon Júnior, Giovanna Ribeiro Souto, Paulo Eduardo Alencar Souza, Martinho Campolina Rebello Horta

**Affiliations:** 1 Oral Pathology Section and Graduate Program in Dentistry, School of Dentistry, Pontifícia Universidade Católica de Minas Gerais (PUC Minas), Belo Horizonte, MG, Brazil; 2 Faculdade Pitágoras de Ipatinga, Ipatinga, MG, Brazil; 3 Centro Universitário de Patos de Minas (UNIPAM), Patos de Minas, MG, Brazil

## Abstract

**Background:**

Oral squamous cell carcinoma (OSCC) is the most prevalent malignant head and neck tumor, excluding the nonmelanoma skin cancer. Despite recent advances in the diagnosis and treatment, the disease's mortality rate is nonetheless high. The presence of isolated neoplastic cells or small clusters of up to four cells at the tumor’s invasive front, named tumor budding, is associated with a worse prognosis in OSCC. Angiogenesis has also been recognized as a determining factor in the progression of malignancies and in the development of metastases. Several studies have investigated the assessment of microvascular density (MVD) as a potential prognostic factor in OSCC. This study aimed to evaluate, in OSCC, differences in MVD between tumors with high-intensity tumor budding and tumors with low-intensity or no tumor budding. In samples with high-intensity tumor budding, differences in MVD between the budding area and the area outside the budding were also evaluated. Moreover, the study assessed differences in MVD concerning clinicopathological characteristics such as sex, age, tobacco smoking, tumor location and tumor size.

**Material and Methods:**

One hundred and fifty [150] samples of OSCC were subjected to immunohistochemistry to assess the intensity of tumor budding (by immunostaining for multi-cytokeratin) and MVD (by immunostaining for CD34 and CD105, independently). The data were treated using descriptive and analytical statistics.

**Results:**

There were no differences in MVD, assessed by immunostaining for CD34 or CD105, concerning clinicopathological characteristics such as sex, age, tobacco smoking, tumor location and tumor size (*p* > 0.05). Tumors with high-intensity tumor budding did not show differences in MVD, assessed by immunostaining for CD34 or CD105, when compared to tumors with low-intensity or no tumor budding (*p* > 0.05). However, in samples with high-intensity tumor budding, the MVD assessed by immunostaining for CD34 was higher in the budding area than in the area outside the budding (*p* < 0.05). This difference was not observed when MVD was assessed by immunostaining for CD105 (*p* > 0.05).

**Conclusions:**

The higher MVD in the budding area may be an additional indication that this is a peculiar region of the tumor, associated with biological phenomena related to tumor progression.

** Key words:**Oral squamous cell carcinoma, tumor budding, microvascular density.

## Introduction

Oral squamous cell carcinoma (OSCC) is the most prevalent malignant head and neck tumor, excluding the nonmelanoma skin cancer (1). Despite recent advances in the diagnosis and treatment, the disease's mortality rate is nonetheless high, with nearly 65% short-term survival rate (2).

A fundamental feature of malignant neoplastic cells is their capacity for local invasion and metastasis. One of the morphological markers related to this ability, named tumor budding, is characterized by the presence of isolated neoplastic cells or small clusters of up to four cells at the tumor invasive front. This phenomenon, considered a morphological expression of the epithelial–mesenchymal transition, has become increasingly relevant in recent years due to its association with adverse clinical and pathological characteristics. Tumor budding is associated with aggressive tumor behavior and a worse prognosis, being a predictor of lymphovascular invasion, lymph node metastasis, and a lower survival rate (2-4).

Angiogenesis, characterized by the formation of new blood vessels, is a fundamental biological phenomenon in processes such as embryogenesis and wound healing. It has also been recognized as a determining factor in the progression of malignancies and in the development of metastases. Several studies have investigated the assessment of microvascular density (MVD), an indicator of angiogenesis, as a potential prognostic factor in different tumors, including OSCC (5,6).

The aim of this study was to evaluate, in OSCC, differences in MVD between tumors with high-intensity tumor budding and tumors with low-intensity or no tumor budding. In samples with high-intensity tumor budding, differences in MVD between the budding area and the area outside the budding were also evaluated. Moreover, the study assessed differences in MVD concerning clinicopathological characteristics such as sex, age, tobacco smoking, tumor location and tumor size.

## Material and Methods

- Sample selection

This study was approved by the Research Ethics Committee of PUC Minas (CAAE 74605417.0.0000.5137). Firstly, we evaluated a total of 260 OSCC samples of the Oral Pathology Laboratory at the PUC Minas School of Dentistry. All of these samples were previously obtained by incisional biopsy for diagnostic purposes, having been fixed in 10% formaldehyde and processed according to routine procedures for inclusion in paraffin. After the evaluation of these 260 samples, the following were eliminated: samples with scarce tumor parenchyma, samples with tumor stroma considered insufficient to assess MVD, and samples containing insufficient material to provide new histological sections. The final count was 150 OSCC samples.

- Clinicopathological data

Clinicopathological characteristics (sex, age, tobacco smoking, tumor location and tumor size) of the 150 OSCC cases were obtained from the Anatomic Pathology Requisition Form. Sex data were available in 150 cases, age data in 143 cases, tobacco smoking data in 106 cases, tumor location data in 150 cases and tumor size data in 120 cases.

- Immunohistochemistry reactions

The immunohistochemistry technique was used to identify 1) multi-cytokeratin AE1 / AE3 (used in the evaluation of tumor budding by identifying neoplastic epithelial cells); 2) CD34 protein (used to assess MVD); and 3) CD105 protein (used to assess MVD). Antigen retrieval was performed using Trilogy (Cell Marque, Rocklin, CA, USA). The following primary antibodies were used: I) anti-multi-cytokeratin (cocktail of clones AE1 and AE3, diluted 1:50 [Biosystems, Newcastle, UK]); II) anti-CD34 (clone QBEnd/10, diluted 1:200 [Medaysis, Livermore, CA, USA]); and III) anti-CD105 (endoglin) (clone EPR19911 – equivalent to clone EP274, diluted 1:50; [Medaysis, Livermore, CA, USA]). The amplification system used was the Reveal Biotin-Free (Spring Bioscience, Pleasanton, CA, USA). As positive controls, samples of pyogenic granuloma (for CD34 and CD105) and fibrous hyperplasia (for multi-cytokeratin AE1 / AE3) were used. Negative control was determined by omission of the primary antibodies, as well as by the use of monoclonal antibodies with the same isotype and different specificity of the primary antibodies employed in the study.

- Evaluation of tumor budding intensity

The evaluation of tumor budding intensity was performed in the 150 samples submitted to the multi-cytokeratin immunostaining (used to identify OSCC cells), as suggested by Leão *et al*. (7). This assessment was made by one experienced examiner (MCRH), using an Olympus BX51 optical microscope with x10 ocular lens and field number 22 (Olympus Optical, Tokyo, Japan).

**Figure 1:**
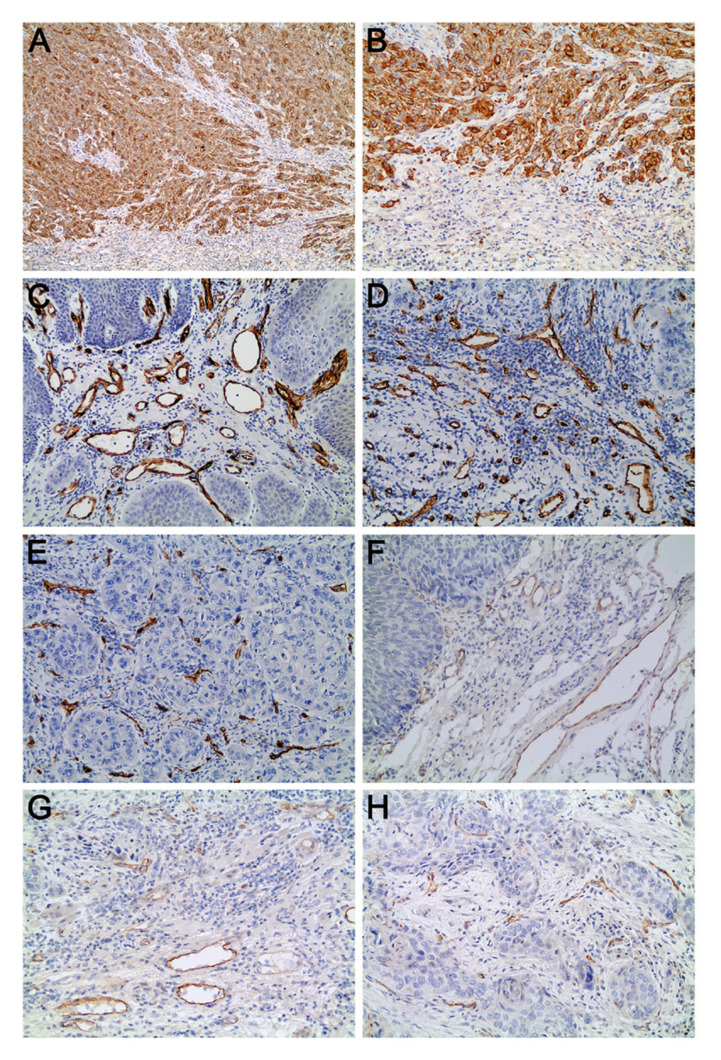
A and B) Oral squamous cell carcinoma (OSCC) showing high-intensity tumor budding (multi-cytokeratin immunostaining; A: 100x; B: 200x); C) Microvessels immunostained for CD34 in OSCC with low-intensity or no tumor budding (CD34 immunostaining, 200x); D) Microvessels immunostained for CD34 in the budding area of OSCC showing high-intensity tumor budding (CD34 immunostaining, 200x); E) Microvessels immunostained for CD34 in the area outside the budding of OSCC showing high-intensity tumor budding (CD34 immunostaining, 200x); F) Microvessels immunostained for CD105 in OSCC showing low-intensity or no tumor budding (CD105 immunostaining, 200x); G) Microvessels immunostained for CD105 in the budding area of OSCC showing high-intensity tumor budding (CD105 immunostaining, 200x); H) Microvessels immunostained for CD105 in the area outside the budding of OSCC showing high-intensity tumor budding (CD105 immunostaining, 200x).

The evaluation was carried out in a single x200 power field, following the criteria proposed by Wang *et al*. (3). Tumor budding was defined as the presence of isolated neoplastic cells or small clusters of up to four cells at the tumor invasive front. Samples were initially evaluated using the lowest power objective lens to select the areas with the highest intensity of tumor budding. Subsequently, using a x20 objective lens, the number of tumor buds (number of isolated neoplastic cells or small clusters of up to four cells) was counted in one single elected x200 power field (the field showing the highest number of tumor buds). The samples were then classified into I) tumors with high-intensity tumor budding, that is, tumors showing five or more tumor buds in one x200 power field (Fig. 1); II) tumors with low-intensity or no tumor budding, that is, tumors showing fewer than five tumor buds or no tumor bud in one x200 power field.

The intra-examiner agreement for assessing the intensity of tumor budding was calculated using Cohen's kappa. For this analysis, the examiner performed estimations on 40 samples, each two times: T1 (initial evaluation) and T2 (30 days later). The value obtained for kappa was 0.95 (95% CI: 0.85 to 1.04), which is an excellent agreement according to the criteria of Cicchetti and Sparrow (8). The concordance analysis was performed by the StatsToDo statistical program at the website www.statstodo.com (StatsToDo Trading, Brisbane, QLD, Australia).

- Evaluation of MVD

MVD was independently evaluated in samples submitted to immunostaining for CD34 and CD105. This evaluation was performed by one experienced examiner (EMA), using the ImageJ software (National Institutes of Health, Bethesda, MD, USA), with images captured by a digital camera attached to an Olympus BX51 optical microscope (Olympus Optical, Tokyo, Japan).

In tumors with high-intensity tumor budding, the evaluation of MVD was carried out in two distinct areas: I) the tumor budding area – one single x200 power field showing the highest intensity of tumor budding and II) the area outside the tumor budding – one single x200 power field, outside the budding area, showing the highest MVD. In tumors with low-intensity or no tumor budding, this evaluation was carried out in one single x200 power field showing the highest MVD.

The following were considered as a single counTable microvessel: 1) one vessel lumen lined by an immunostained endothelium; 2) one immunostained endothelial cell; or 3) one cluster of immunostained endothelial cells clearly separated from adjacent clusters. Visible lumen or the presence of red blood cells in the lumen were not considered as criteria to define a counTable microvessel (9).

Afterward, the variable MVD was generated as follows: MVD = number of microvessels in a x200 power field.

The intra-examiner agreement for the evaluation of MVD was calculated by the intraclass correlation coefficient (ICC), independently assessed for MVD by CD34 and CD105. For this analysis, the examiner performed estimations on 50 samples at two separate times, T1 (initial evaluation) and T2 (30 days later). The values obtained for the ICC were 0.97 assessed by CD34 immunostaining and 0.92 assessed by CD105 immunostaining, respectively. These represent an excellent agreement according to the criteria of Cicchetti and Sparrow (8). The concordance analysis was performed by the StatsToDo statistical program (StatsToDo Trading, Brisbane, QLD, Australia).

- Statistical analysis

**Figure 2:**
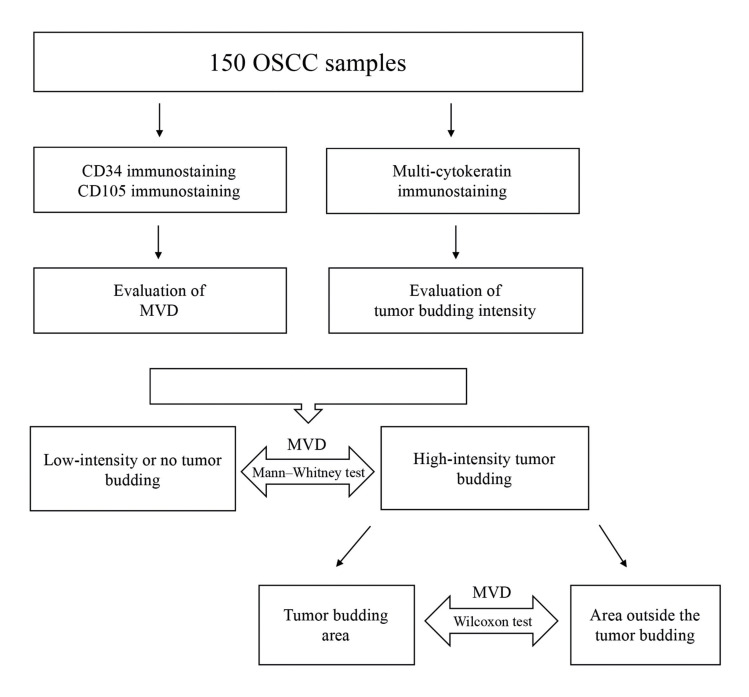
Study design and statistical analyses. The Mann–Whitney test was used to assess differences in MVD between tumors with high-intensity tumor budding and tumors with low-intensity or no tumor budding. In tumors with high-intensity tumor budding, the Wilcoxon test was performed to evaluate differences in MVD between the budding area and the area outside the budding. Note: OSCC: oral squamous cell carcinoma; MVD: microvascular density.

The D’Agostino–Pearson normality test demonstrated that MVD data showed a non-normal distribution. The Mann–Whitney test was then used to assess differences in MVD, independently for CD34 and CD105, between tumors with high-intensity tumor budding and tumors with low-intensity or no tumor budding. In tumors with high-intensity tumor budding, the Wilcoxon test was performed to assess differences in MVD between the budding area and the area outside the budding, independently for CD34 and CD105. The Mann–Whitney test as well as the Kruskal–Wallis test followed by the Dunn’s multiple comparisons test were used to assess differences in MVD, independently for CD34 and CD105, concerning clinicopathological characteristics (sex, age, tobacco smoking, tumor location and tumor size). The level of significance was set at 5%. Analyses were performed using GraphPad Prism software (San Diego, CA, USA). The study design and the statistical analyses are demonstrated in Fig. 2.

## Results

One hundred and fifty [150] samples of OSCC were evaluated. The Table 1 shows the clinicopathological characteristics of the OSCC cases, such as sex, age, tobacco smoking, tumor location and tumor size. In regard to sex (data available in 150 cases), the sample consisted of 116 men (77.3%) and 34 women (22.7%). The mean and standard deviation of the age of the patients (data available in 143 cases) were 58.90 13.14 years (the minimum age was 30 years, and the maximum age was 94 years). In relation to tobacco smoking (data available in 106 cases), there were 61 (57.5%) current smokers, 23 (21.7%) former smokers and 22 (20.8%) never smokers. Tumors from different anatomical locations were evaluated (data available in 150 cases): tongue (*n*=64; 42.7%), floor of the mouth (*n*=24; 16.0%), alveolar ridge (*n*=19; 12.7%), lip (*n*=17; 11.3%) and other locations (*n*=26; 17.3%). Finally, the tumor size (data available in 120 cases) was categorized as ≤ 2 cm in 54 cases (45%), as > 2 and ≤ 4 cm in 43 cases (35.8%) and as > 4 cm in 23 cases (19.2%).

There were no differences in MVD (assessed by CD34 immunostaining or by CD105 immunostaining) concerning clinicopathological characteristics such as sex, age, tobacco smoking, tumor location and tumor size (*p* > 0.05; Table 1).

The evaluation of tumor budding of the 150 OSCC samples revealed that 83 samples (55.4%) showed high-intensity tumor budding (Fig. 1), whereas 67 samples (44.6%) showed low-intensity or no tumor budding.

There was no difference in MVD (assessed by CD34 immunostaining or by CD105 immunostaining) between tumors with high-intensity tumor budding and tumors with low-intensity or no tumor budding (*p* > 0.05; Tables 2 and 3; Fig. 1). However, in tumors with high-intensity tumor budding, the MVD assessed by CD34 immunostaining was higher in the budding area than in the area outside the budding (*p* < 0.05; Table 2; Fig. 1). This difference was not observed when the MVD was assessed by CD105 immunostaining (*p* > 0.05; Table 3; Fig. 1).

**Table 1:**
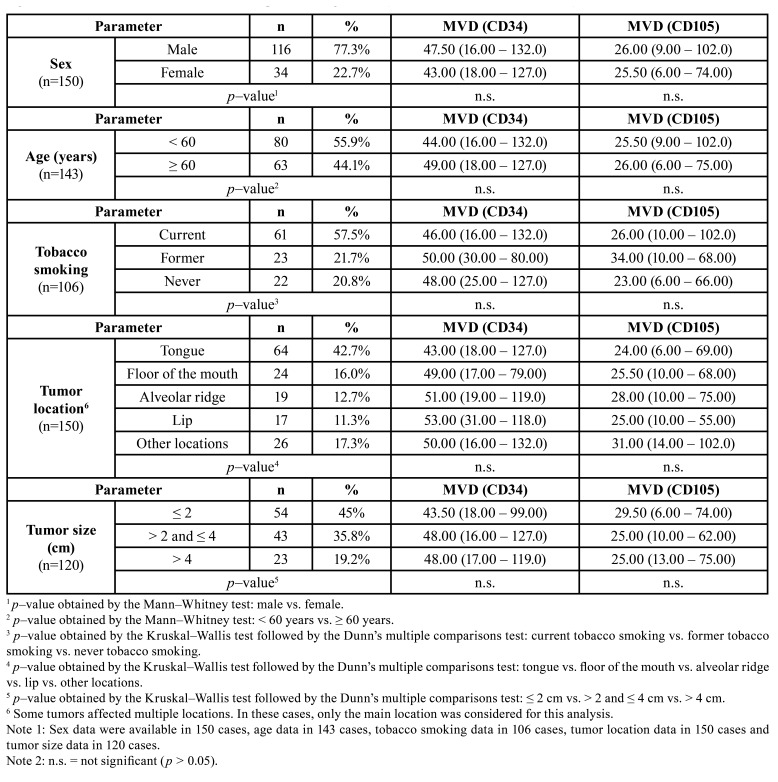
Clinicopathological characteristics of the oral squamous cell carcinoma (OSCC) cases and evaluation of differences in microvascular density (MVD) concerning sex, age, tobacco smoking, tumor location and tumor size. The MVD was independently evaluated by immunostaining for CD34 and CD105. The MVD data was expressed using median (minimum value – maximum value).

**Table 2:**
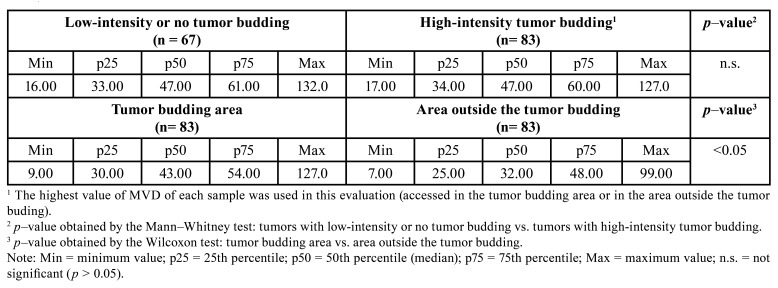
Data and comparisons of microvascular density (MVD) in oral squamous cell carcinoma (OSCC) (assessed by immunostaining for CD34).

**Table 3:**
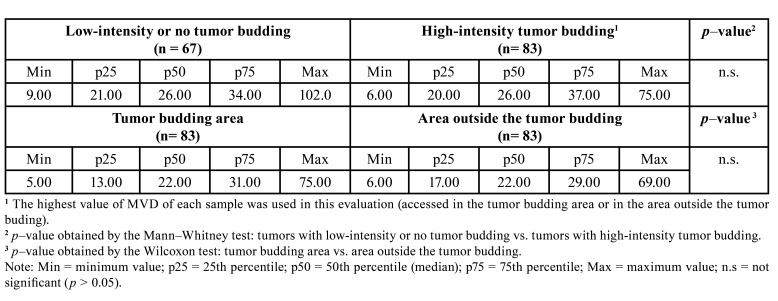
Data and comparisons of microvascular density (MVD) in oral squamous cell carcinoma (OSCC) (assessed by immunostaining for CD105).

## Discussion

OSCC, a tumor with high rates of metastasis, shows a complex and abundantly vascularized stroma. The study of biomarkers such as MVD and tumor budding increases the knowledge concerning the biological behavior of this tumor, with prognostic and therapeutic implications.

The evaluation of MVD has been applied in the prognostic prediction of several human tumors, with variable results. Specific endothelial antibodies, such as anti-CD105 and anti-CD34, appear to be more accurate for this purpose. Endoglobin (CD105) and CD34 have been associated with the proliferation of endothelial cells and are considered important markers of neovascularization in solid malignant tumors (5,6,10-13).

The present study showed no difference in MVD in OSCC, assessed by both CD34 and CD105 immunostaining, between tumors with high-intensity tumor budding and tumors with low-intensity or no tumor budding. To the best of our knowledge, there are no studies in the English literature evaluating association between angiogenesis and tumor budding. Our results could indicate that, even though both of these parameters are prognostic factors in OSCC, they are not necessarily related. Despite the absence of studies considering tumor budding and angiogenesis, several authors have evaluated MVD (through immunostaining for CD34 and CD105) and clinical-pathological parameters of tumor aggressiveness. Some studies have evaluated MVD in OSCC and observed no differences in this parameter between well, moderately and poorly differentiated tumors (11). Nevertheless, other studies have shown that the MVD in OSCC was higher in less differentiated tumors (13). Shao *et al*. (14) demonstrated an association between higher MVD in OSCC and lower survival. However, Toyoda *et al*. (15) demonstrated that MVD in OSCC is not related to survival. Miyahara *et al*. (16) observed that the MVD in OSCC was higher in tumors with regional metastases and a more infiltrative histological invasion pattern. Nevertheless, these authors showed no difference in MVD between well, moderately and poorly differentiated tumors. Chuang *et al*. (17) observed that MVD in OSCC was positively associated with tumor stage (TNM), regional metastases, tumor necrosis, tumor thickness, and perineural invasion. De Sousa *et al*. (18) demonstrated that the proliferation of microvessels, immunostained for CD105, increases the occurrence of regional metastases in OSCC.

In our study, in tumors with high-intensity tumor budding, the MVD (assessed by CD34 immunostaining) was higher in the budding area than in the area outside the budding. This result may be another indication that the invasive front and, specifically, the area of tumor budding, is a peculiar region of the tumor, associated with biological phenomena related to tumor progression. One of the most promising findings in OSCC histological grading was the recognition of the importance of the characteristics of the deepest and most invasive areas of ​​the tumor—named as the invasive front—in determining its biological aggressiveness. An increasing amount of evidence supports the hypothesis that morphological and molecular characteristics of the tumor invasive front are of great importance for the prognosis of OSCC (19). In this context, and in line with these results, our research group has already demonstrated that the tumor budding area, when compared to the area outside the budding, has a higher rate of cell proliferation (20) and a higher expression of cancer stem cell-like marker ALDH1 (21), reinforcing the hypothesis that tumor budding is a phenomenon associated with the biological behavior of the OSCC, especially that related with tumor progression and metastasis.

Epithelial–mesenchymal transition (EMT) is a dynamic multi-stage cellular phenomenon in which epithelial cells lose their cell–cell adhesion and gain migratory and invasive characteristics typical of mesenchymal cells. Activation of EMT is considered an important marker of the process of local invasion and metastasis of malignant tumors. EMT involves the loss of the epithelial marker E-cadherin (a key cell–cell adhesion molecule in epithelial cells) and an increase in the levels of one or more mesenchymal markers, such as vimentin (4,22).

Tumor budding is considered the morphological expression of EMT (4,23), representing two essential properties for local invasion and metastasis of malignant neoplasms, namely loss of cell cohesion and active invasive movement (3,24,25). In this context, several studies have shown a positive association between high-intensity tumor budding and worse prognosis in OSCC (2,24,26). It is worth noting that the evaluation of tumor budding in OSCC through immunostaining for multi-cytokeratin, a method performed in the present study, is supported by the literature. In fact, a recent study published by our research group showed that immunostaining for multi-cytokeratin for tumor budding evaluation has higher reproducibility, higher repeatability, and less difficulty than hematoxylin and eosin staining (7).

As opposed to what was observed when CD34 immunostaining was employed, in tumors with high-intensity tumor budding, we did not observe differences in MVD, assessed by CD105 immunostaining, between the budding area and the area outside the budding. There are divergences in the literature on the ideal blood microvessel marker. CD34 is a good indicator of the maturation of immature vessels, being expressed in all proliferating or not endothelial cells. CD105 is expressed exclusively in proliferating endothelial cells (10,12). Markers such as CD34 are pan-endothelial and expressed in vessels of normal and neoplastic tissue. CD105 is a marker of endothelial cells expressed in the process of tumor angiogenesis but scarce or absent in normal tissues. Currently, CD105 has been suggested as an important and specific marker to determine angiogenesis and its role in determining the prognosis of OSCC. It is suggested that CD105 is an angiogenic marker induced by hypoxia and is therefore expressed especially in solid tumors where this biological phenomenon is relevant (27). Still in this context, CD34 has been considered by some authors as a superior angiogenic marker when compared to other markers, generating better technical results, with less background, which would facilitate the evaluation of the immunohistochemistry reaction (6). This discussion could explain the divergent results of MVD observed in the present study.

Finally, no differences in MVD (assessed by CD34 immunostaining or by CD105 immunostaining) were observed concerning clinicopathological characteristics such as sex, age, tobacco smoking, tumor location and tumor size, suggesting that MVD in OSCC is not influenced by these parameters.

Our study has some limitations. First, some clinical data is missing such as staging and patient survival. Second, it contains no evaluation of other morphological markers that may have an impact on prognosis, including histological differentiation, tumor thickness, invasion pattern, depth of invasion, or lymphovascular invasion. Third, the study used OSCC samples from different anatomical locations, associated with different etiopathogeneses.

The use of incisional biopsy samples could, in principle, be considered another limitation of our study, since the areas with high-intensity tumor budding are not necessarily included in samples collected by incisional biopsy. However, several studies have demonstrated the viability of using incisional biopsies for the evaluation of tumor budding in OSCC. Almangush *et al*. (28) showed that the tumor budding scores of preoperative OSCC biopsies were statistically associated with the scores of postoperative tumor resection samples. Seki *et al*. (29) showed that the evaluation of tumor budding in samples of incisional biopsy of OSCC is an independent prognostic factor, recommending that the evaluation of tumor budding in preoperative incisional biopsies of OSCC should be considered in routine histological evaluations as a new criterion for the decision to perform prophylactic neck dissection. It should also be noted that several studies on the evaluation of tumor budding in OSCC have been carried out adequately in samples of incisional biopsy (7,20,21,29).

In this context, a very important topic must be highlighted. The future use of tumor budding assessment as a determinant indicator of the need for prophylactic neck dissection necessarily involves the analysis of preoperative samples obtained through incisional biopsy. These considerations are in line with the results of the systematic review recently published by Almangush *et al*. (30), which observed a strong association between the tumor budding score in diagnostic incisional biopsies and the corresponding surgical samples (obtained later by total tumor resection). Moreover, according to these authors, the evaluation of tumor budding using samples obtained by incisional biopsies showed prognostic value for both regional metastases and patient survival.

In conclusion, our study demonstrated no differences in MVD, assessed by both CD34 and CD105 immunostaining, in OSCC with high-intensity tumor budding when compared to tumors with low intensity or no tumor budding. Nevertheless, in samples with high-intensity tumor budding, the MVD assessed by CD34 immunostaining was higher in the budding area than in the area outside the budding. This difference was not observed when MVD was assessed by CD105 immunostaining. The higher MVD in the budding area may be an additional indication that this is a peculiar region of the tumor, associated with biological phenomena related to tumor progression.
